# Ambient pressure ammonia decomposition using Ga–Co supported catalytically active liquid metal solutions

**DOI:** 10.1039/d6cy00362a

**Published:** 2026-06-06

**Authors:** Philipp Rothgängel, Nicola Taccardi, Aaron Luke Folkard, Jakob Söllner, Alexander Søgaard, Andreas Körner, Andreas Hutzler, Matthias Thommes, Marco Haumann, Peter Wasserscheid

**Affiliations:** a Helmholtz-Institute Erlangen-Nürnberg for Renewable Energy (IET-2), Forschungszentrum Jülich GmbH (FZJ) Cauerstraße 1 Erlangen 91058 Germany p.wasserscheid@fz-juelich.de; b Institute for a sustainable Hydrogen Economy (IHE), Forschungszentrum Jülich GmbH (FZJ) Brainergy Park Jülich, An der Deutschen Welle 7a 52428 Jülich Germany; c Lehrstuhl für Chemische Reaktionstechnik (CRT), Friedrich-Alexander-Universität Erlangen-Nürnberg (FAU) Egerlandstr. 3 Erlangen 91058 Germany nicola.taccardi@fau.de peter.wasserscheid@fau.de; d School of Chemistry and Physics, University of KwaZulu-Natal Durban KwaZulu Natal South Africa; e Lehrstuhl für Thermische Verfahrenstechnik (TVT), Friedrich-Alexander-Universität Erlangen-Nürnberg (FAU) Egerlandstr. 3 Erlangen 91058 Germany; f Research Centre for Synthesis and Catalysis, Department of Chemistry, University of Johannesburg P.O. Box 524, Auckland Park 2006 South Africa

## Abstract

Ga–Co supported catalytically active metal solutions (SCALMS) on silicon carbide (SiC) were successfully tested for ambient pressure ammonia decomposition in the temperature range between 480 and 580 °C. Comparison studies of a Ga_59_Co SCALMS system with their monometallic equivalents Co/SiC and Ga/SiC revealed a massively enhanced ammonia decomposition activity of the supported alloy catalyst. The Ga_59_Co/SiC outperformed a monometallic benchmark Co/SiC catalyst in terms of the Co specific H_2_ productivity by up to one order of magnitude. Monometallic Ga/SiC, in contrast, showed no activity for ammonia decomposition up to 580 °C. We report productivities of 487 g_H_2__ g_Co_^−1^ h^−1^ at 5% conversion and 550 °C at a weight hourly space velocity (WHSV) of 29 000 mL_N_ g_cat_^−1^ h^−1^ for our SCALMS system. Apparent activation energies (*E*_A,app_) determined from temperature variation experiments give no indication for diffusion limitations under the applied reaction conditions. Therefore, we present a new catalyst material concept for ammonia decomposition with high potential for practical and technical application.

## Abstract 2

Ga–Co SCALMS catalysts enable highly efficient ammonia decomposition, increasing cobalt-specific hydrogen productivity by one order of magnitude.

## Graphical abstract

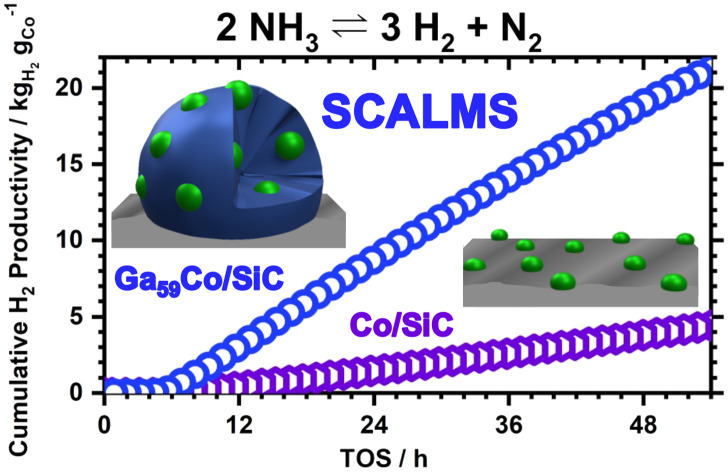

## Introduction

In pursuit of decarbonisation, various nations have announced strategies and formed alliances focusing on hydrogen (H_2_) as a renewable energy carrier, given its high gravimetric energy density (33.3 kWh kg^−1^) and environmentally benign properties. However, large-scale storage and long-distance transportation present technical challenges due to hydrogen's low volumetric energy density (3 kWh m^3^ at 20 °C and 1 bar) and small molecular size.^1–5^

Green and/or blue ammonia (NH_3_) has been proposed as a promising H_2_ carrier to address these limitations. Due to its high volumetric H_2_ storage capacity (∼108 kg_H_2__ m^3^) and ease of liquefaction (*e.g.* 25 °C, 10 bar), it is a potentially cost-efficient alternative for long-distance transportation.^3,6–8^ The chemically bound H_2_ can be released on demand through thermal cracking, producing only nitrogen (N_2_) as a byproduct, as shown in eqn (1).

1 2NH_3_ ⇌ N_2_ + 3H_2_; Δ*H*^0^_298_ = 92.44 kJ mol^−1^

Using a catalyst to enhance the efficiency of hydrogen release is essential to optimise the overall net energy balance, given that the reaction requires temperatures above 500 °C due to its endothermic nature. The reaction is characterized by adsorption and desorption steps (eqn (2), (6), and (7)) and the cleavage of N–H bonds (eqn (3), (4), and (5)). N–H cleavage and the recombintaion of two adsobred nitrogen N_ad_ (eqn (7)) are the two main rate determining steps reported.^9,10^ Enhanced electron transfer, electron donating additives, and structures or morpholgies allowing enhanced N recombination are ways to adress these topics. The investigation of such new, improved, and stable catalyst systems for thermal NH_3_ decomposition is therefore of high interest to current research and industry.

2 NH_3,g_ + * ⇌ NH_3,ad_

3 NH_3,ad_ + * ⇌ NH_2,ad_ + H_ad_

4 NH_2,ad_ + * ⇌ NH_1,ad_ + H_ad_

5 NH_1,ad_ + * ⇌ N_ad_ + H_ad_

6 2H_ad_ ⇌ H_2,g_ + 2*

7 2N_ad_ ⇌ N_2,g_ + 2*

Supported catalytically active liquid metal solutions (SCALMS) represent such a new class of heterogeneous catalysts. The SCALMS concept has recently attracted a lot of interest for the dehydrogenation of propane, butane, heptane, and methylcyclohexane as well as for the oligomerization of ethene.^11–20^ First approaches in ammonia synthesis have also been reported.^21^ Conceptually, SCALMS are composed under reaction conditions of liquid droplets of a catalytically active metal alloy on a porous support. Those alloys consist typically of a low-melting metal, such as, *e.g.*, Ga, that serves as matrix for dissolving smaller amounts of a catalytically active metal. Precious metals, like Pt, Pd or Rh, and Ni as non-precious metal have been utilized as active component of SCALMS systems so far.^11–14,20^ A unique feature of SCALMS systems is that the catalytic reaction occurs at dynamically emerging single-atom active sites at the gas–liquid interface. This single-atom nature of the atomically-dispersed active metal has been demonstrated for precious metal based SCALMS systems by spectroscopic, microscopic, and theoretical studies.^11,22–24^

The scope of this study is the initial investigation of SCALMS catalysts and their general suitability for thermal NH_3_ decomposition by combining commonly reported metals, such as Co, Ni, and Cu, with Ga.^14–19^ A comparison between the monometallic and bimetallic supported catalysts for a selected active metal aims to provide initial insights into the feasibility and attractiveness of the SCALMS concept for ammonia cracking.

## Experimental

### Preparation of catalysts

For our study, we prepared the Ga–X (active metal X: Co, Ni, or Cu) on SiC materials as well as monometallic Ga on SiC and Co on SiC materials (for comparative investigations) by a combination of ultrasonication-based emulsion preparation, wet impregnation and galvanic displacement. A similar methodology had been applied by our group previously to prepare Ga–Pt and Ga–Ni SCALMS materials.^12,15,20^ The galvanic replacement reaction utilizes the standard reduction potentials of Ga, Co, Ni and Cu in aqueous solution (*E*_Ga^3+^/Ga^0^_ = −0.53 V, *E*_Co^2+^/Co^0^_ = −0.28 V, *E*_Ni^2+^/Ni^0^_ = −0.25 V, and *E*_Cu^2+^/Cu^0^_ = +0.34 V) to ensure the deposition of the secondary metals (higher potential) on Ga droplets (lowest potential) as described previously.^25–30^ The SiC support is expected to not participate in the reaction due to its inertness in water and its notable stability, even in the presence of HF.

The benchmark Co/SiC material was prepared using incipient wetness impregnation and calcination at 600 °C for 3 h (heating ramp 2 °C min^−1^, dried at 150 °C for 2 h). An alternatively higher loaded Co/SiC catalyst was prepared using the same method. All details regarding the catalyst material preparation are shown in the SI.

### Characterization of fresh and spent catalysts

The metal weight loadings *w*_metal_ and the Ga to active metal X atomic ratio (Ga_*n*_X) have been determined using inductively coupled plasma atomic emission spectroscopy (ICP-AES). Further characterizations of the used materials were carried out using N_2_ sorption, Hg porosimetry, as well as scanning electron and scanning transmission electron microscopy *via* energy-dispersive X-ray spectroscopy (SEM–EDXS and STEM–EDXS). All technical details are given in the SI.

### Catalytic testing

All catalytic experiments were performed in a high-temperature set-up consisting of a tubular split furnace (Nabertherm) with one heating zone of 250 mm and an isothermal zone of 80 mm. Inert quartz tubes (*L*: 1000 mm, OD: 13 mm, ID: 9.8 mm) with three pins at a height of 555 mm from the bottom end to support the catalyst bed were used as reactors. An additional quartz capillary (*L*: 700 mm, OD: 1.6 mm, DI: 0.8 mm) containing thermocouples to determine the catalyst bed temperature was placed within the catalyst bed from the top. A total of 1.0 g of catalyst was placed on top of 0.3 g quartz wool, and 0.3 g activated carbon spheres (200 μm) to ensure a uniform gas flow. All catalysts were reduced *in situ* in a H_2_ : N_2_ mixed gas flow (molar ratio of 3 : 1) at 585 °C to remove the oxide skin on the metal droplets. The reduction of gallium oxide in the presence of hydrogen and a hydrogen-activating metal has been described in several publications.^15,31–33^ After this catalyst preformation procedure, the gas flow was switched to NH_3_, which marks the point zero of the time on stream (TOS). The temperature was stepwise increased to 582 °C in the reactor (gray bar for the first 6 h TOS in Fig. 2). The startup behavior in each experiment was observed at a flow rate of 10 l_N_ h^−1^ undiluted NH_3_ resulting in a weight hourly space velocity (WHSV) of 10 l_N_ g_cat_^−1^ h^−1^ and at temperatures of 582 °C at atmospheric pressure. Follow-up experiments were conducted by changing temperature and gas flow. The off-gas was quantitatively analyzed in a micro gas chromatograph (Micro GC Fusion, INFICON GmbH) with two sample lines and thermal conductivity detectors (TCD), one sample line for permanent gases (carrier gas: Ar) and the other one for ammonia (carrier gas: H_2_). All gases used had a purity of ≥99.999% (supplied by Air Liquide Deutschland GmbH).

## Results and discussion

### Catalyst characterization

Three metals, namely Co, Ni, and Cu, were selected as active metals for the Ga-based SCALMS materials tested in our ambient pressure ammonia decomposition experiments.^9,21,34–37^ These SCALMS materials were prepared *via* impregnation of pre-synthesized, gallium-decorated β-SiC. Analysis of the metal content resulted in Ga loadings of 3.10–6.12 wt%, active metal loadings of 0.04–0.08 wt%, and Ga/active metal ratio values between 47 and 71 (Table 1). According to the corresponding bimetallic phase diagrams, all of these Ga-rich alloys are expected to be present in the liquid state at temperatures exceeding 500 °C.^20,38–40^ On β-SiC supported monometallic Ga (prepared by ultrasonication) and Co (prepared by incipient wetness impregnation) were used for comparison studies. Therefore, the metal loadings aimed to be within the same range as for the SCALMS materials (Table 1).

**Table 1:** Chemical composition of the supported catalytically active liquid metal solutions (SCALMS) and monometallic catalysts prepared

Catalyst	Metal loading (after calcination)/wt%		Molar ratio
	Ga	Active metal	
Ga_59_Co/SiC	3.12	0.04	59
Ga_47_Ni/SiC	4.60	0.08	47
Ga_71_Cu/SiC	6.12	0.08	71
Ga/SiC	4.27	—	—
Co/SiC	—	0.05	—
Co/SiC	—	1.05	—

N_2_ sorption and Hg porosimetry for the utilized SiC supports and functionalized catalysts revealed mainly meso and macro pores in accordance with supplier data (see Fig. S2 to S5 and Table S1).^41^ No significant changes to the pore structure and surface areas due to the catalyst preparation methods were observed. The SCALMS materials before and after reaction were characterized using scanning electron microscopy with elemental mapping *via* energy-dispersive X-ray spectroscopy (SEM–EDXS). This method was employed to evaluate the morphology of the Ga-rich droplets on the external surface of the SiC support material and the distribution of the active metal.

**Fig. 1:**
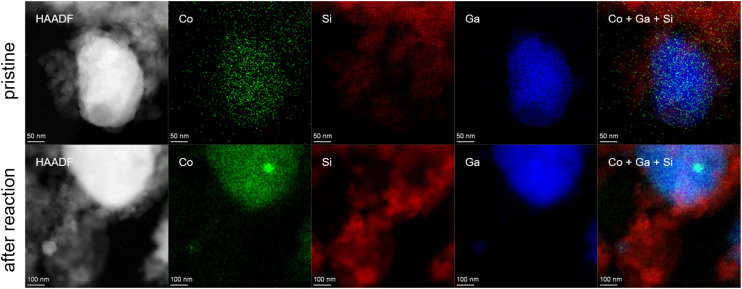
STEM–EDX images of a Ga_59_Co/SiC catalyst before (as-prepared) and after use in the ammonia decomposition reaction. The images show that Co signals are associated with Ga; after use in NH_3_ decomposition, the formation of a Co cluster is observed.

Using ultrasonic emulsification, the Ga droplets were produced dispersed in isopropanol from elemental Ga and directly deposited on the substrate.^12,16^ In accordance with previous works, we found after subsequent galvanic displacement using a cobalt(ii) nitrate hexahydrate solution in 20_v/v_% water in isopropanol, that the alloy droplets were mainly located on the outer surface of the support particles.^12,16,42^ Some alloy droplets were also found at the walls or in cavities larger than 10 μm. The inner pore system did not contain any observable quantities of Co–Ga (see Fig. S8). Unfortunately, the low concentrations of active metals resulted in low signal-to-noise ratios for the SEM-EDXS elemental maps. Therefore, scanning transmission electron microscopy *via* energy-dispersive X-ray spectroscopy (STEM–EDXS) with higher resolution was applied to study the Ga–Co catalysts. Fig. 1 shows images and elemental mapping for the Ga_59_Co/SiC SCALMS as-prepared and after use in catalysis. In both samples the Ga-droplets were always associated with Co-signals. This was expected due to the galvanic displacement of Co on the Ga droplets during preparation. After use in catalysis, the formation of a Co-rich cluster of about 45 nm was observed within the Ga droplet. This may indicate some redistribution of the bimetallic phase and phase separation within the droplet. This may have occurred during the cool-down of the reactor at the end of the experiment. Further microscopic images (including monometallic Co/SiC) are found in the SI (Fig. S6 and S7). This phenomenon has been reported on multiple occasions in the context of liquid metal systems following cooling and subsequent resolidification. Zuraiqi *et al.* observed a Cu-rich Cu–Ga intermetallic particle that was embedded in a Ga droplet. Upon heating the sample to the reaction temperature, complete dissolution of the Cu–Ga particle in Ga was observed.^43^ In their studies on Ga–Ni and Ga–Pt SCALMS systems, Søgaard *et al.* and Madubuko *et al.*, respectively, demonstrated the existence of intermetallic species by means of spectroscopy and DFT simulations, that dissolve completely or partially in the surrounding liquid Ga matrix. The presence of single metal atoms has been observed and predicted to appear at the surface of the liquid metal droplets, while potential solid or amorphous intermetallics submerge in the bulk.^29,44^ It is reasonable to hypothesize that the Ga-Co system behaves in a similar way.

Characterization of the Co-containing samples by XRD revealed in the as-prepared and used state no evidence for the formation of solid intermetallic Ga–Co species. It should be noted that the Co signals were in general low even for high Co loadings so that low signal-to-noise ratios were found in most samples (see Fig. S9 for more details).

### Catalytic screening

**Fig. 2:**
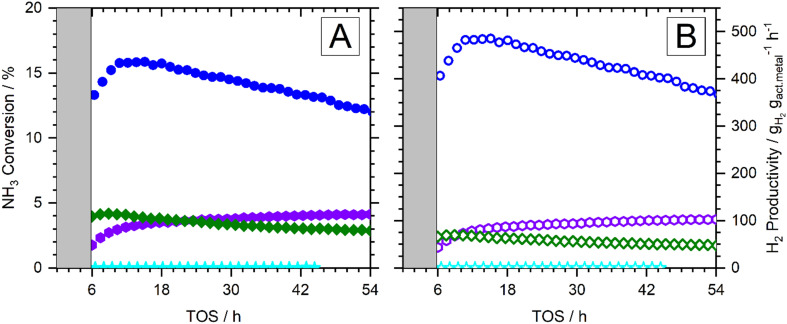
NH_3_ conversion (Fig. 2A, filled symbols) and H_2_ productivity specific to the active metal mass (Fig. 2B, hollow symbols) over 54 h time on stream (TOS) for four different ammonia decomposition catalysts at equal reaction conditions: SCALMS Ga_59_Co/SiC (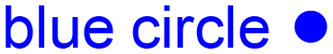, *m*_cat_ = 1.00 g, *V*_cat_ = 1.8 ml, *w*_Co_ = 0.04 wt%, *w*_Ga_ = 3.12 wt%); Ga_47_Ni/SiC (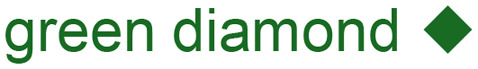, *m*_cat_ = 1.00 g, *V*_cat_ = 1.5 ml, *w*_Ni_ = 0.04 wt%, *w*_Ga_ = 3.12 wt%); Ga_71_Cu/SiC (
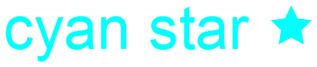
, *m*_cat_ = 1.00 g, *V*_cat_ = 1.9 ml, *w*_Cu_ = 0.08 wt%, *w*_Ga_ = 6.12 wt%), and monometallic Co/SiC (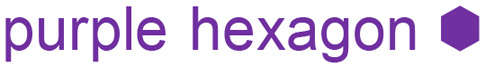, *m*_cat_ = 1.00 g, *V*_cat_ = 1.5 ml, *w*_Co_ = 0.05 wt%). Reaction conditions: *T*_cat_ = 582 °C, *p*_total_ = 1 bar(a), WHSV = 10 l_N_ g_cat_^−1^ h^−1^. The figures show every 36th data point for clarity.

The performance of the three SCALMS systems in NH_3_ decomposition was evaluated at 582 °C and under atmospheric pressure in a quartz tube fixed-bed reactor using an undiluted NH_3_ feed stream. The blank quartz tube reactor and monometallic Ga/SiC displayed negligible conversion levels in the respective blank experiments (see Fig. S10). Of the three metals studied, the presence of Co within the liquid Ga matrix (*i.e.*, the Ga_59_Co/SiC SCALMS catalyst) resulted in the highest conversion of NH_3_ (Fig. 2A). No activity was observed for Ga_71_Cu/SiC SCALMS, while the Ga_47_Ni/SiC SCALMS showed conversions below 5.0%. The Ga–Co SCALMS system showed an activation period of 13 h TOS and reached at this time a conversion level of 15.9 ± 0.1%, followed by slow deactivation to reach a conversion of 12.1 ± 0.1% after 54 h TOS. For the Ga–Ni SCALMS system under investigation the initial activation period was around 10 h TOS, after which the system reached an ammonia conversion of 4.2 ± <0.1% followed also by slow deactivation. The Ga–Ni system reached 2.9 ± <0.1% after 54 h TOS. The observed initial activation phase can be explained by the reduction of a thin, passivating GaO_*x*_ skin on the alloy droplets that remains even after H_2_ pretreatment, as previously described by our group.^20,29^ The passivating skin is further reduced by activated hydrogen during the ammonia decomposition induced by active species, such as Co and Ni.^12,20,30^ We hypothesize that the deactivation of the SCALMS systems is mainly caused by trace water impurities within the supplied ammonia (H_2_O ≤ 5 vol. ppm), that accumulated on the SCALMS system.^12,45,46^ Fig. S12 in the SI shows an extended TOS (>330 h) experiment for a Ga_59_Co SCALMS, indicating that the system reached a steady-state after the initial deactivation phase. Furthermore, it was observed that water was expelled from the system during the repetitive flushing process with H_2_/N_2_ mixtures, H_2_, N_2_ or Ar.

Based on these screening results, we focused our further investigations on the Ga_59_Co SCALMS system. To elucidate the promoting effect of dissolving Co into liquid Ga, we studied a monometallic Co/SiC catalyst (0.05 wt% Co) prepared by incipient wetness impregnation under identical conditions. The catalytic results are also shown in Fig. 2. Like the Ga–Co SCALMS, the Co/SiC catalyst shows an activation phase in the first 13 h TOS followed by a flattened further increase in ammonia conversion. The conversion increased from 3.2 ± <0.1% after 13 h TOS to 4.1 ± <0.1% after 54 h TOS. Remarkably, also after 54 h TOS, the ammonia conversion of the monometallic Co/SiC catalyst was about three times lower than that if the Ga_59_Co SCALMS system after 54 h TOS and about four times lower comparing the maximum conversions of both catalyst systems. This impressively supports our hypothesis that the special SCALMS nature of Ga_59_Co SCALMS leads to a very significant activity boost in ammonia decomposition.

Fig. 2B shows the specific H_2_ productivities per mass of active metal. These values allow us to compare the level of metal utilization and the specific activity of each active metal in NH_3_ decomposition. Herein, the Ga_59_Co SCALMS system shows a metal specific activity of 486 g_H_2__ g_Co_^−1^ h^−1^, which is close to seven times higher than the 70 g_H_2__ g_Ni_^−1^ h^−1^ found for the Ga–Ni system. The monometallic Co on SiC showed productivities up to 103 g_H_2__ g_Co_^−1^ h^−1^.

### Influence of SCALMS on cobalt activity

**Fig. 3:**
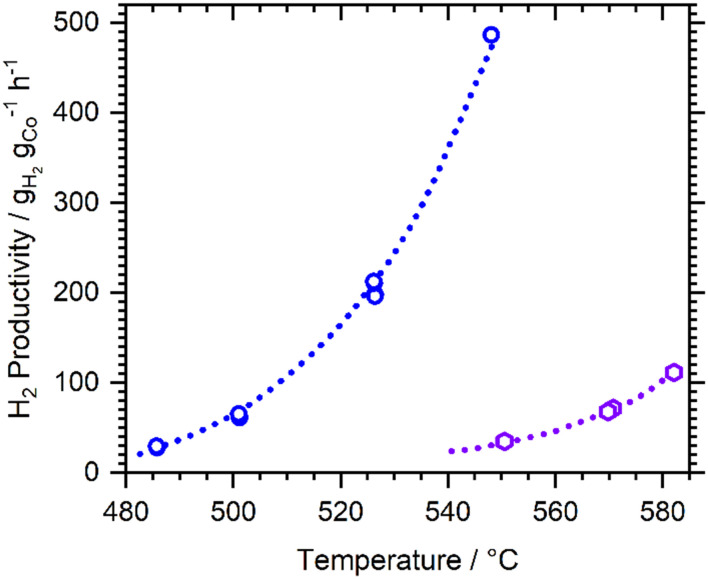
H_2_ productivity specific to the Co mass at 5 ± 0.5% NH_3_ conversion at different catalyst temperatures for Ga_59_Co/SiC (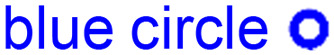, *m*_cat_ = 1.00 g, *V*_cat_ = 1.8 ml, *w*_Co_ = 0.04 wt%, *w*_Ga_ = 3.12 wt%, WHSV = 2–29 l_N_ g_cat_^−1^ h^−1^, *p*_total_ = 1 bar(a)) and Co/SiC (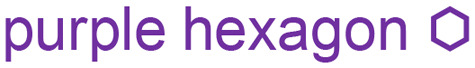, *m*_cat_ = 1.00 g, *V*_cat_ = 1.5 ml, *w*_Co_ = 0.05 wt%, WHSV = 3–9 l_N_ g_cat_^−1^ h^−1^, *p*_total_ = 1 bar(a)). Dotted lines to guide the eye.

In a next set of experiments, we studied the hydrogen productivity in ammonia cracking as a function of temperature comparing the Ga_59_Co SCALMS system and the monometallic Co/SiC catalyst. These experiments were carried out in the temperature range between 485 and 550 °C for Ga_59_Co SCALMS and between 550 and 582 °C for Co/SiC, respectively. Note, that these experiments were carried out at varying NH_3_ flow rates between 30 and 450 ml_N_ min^−1^ in order to adjust an equal conversion level of 5 ± 0.5% independent on the applied reaction temperature. The resulting Co-specific H_2_ productivities as a function of catalyst bed temperatures are shown in Fig. 3.

Apart from the much higher Co-specific hydrogen productivity for the SCALMS system, these experiments revealed a significantly stronger temperature dependency of Ga_59_Co/SiC SCALMS compared to the Co/SiC catalyst. Ga_59_Co/SiC SCALMS showed an exponential productivity increase from 30 g_H_2__ g_Co_^−1^ h^−1^ at 486 °C up to 487 g_H_2__ g_Co_^−1^ h^−1^ at 548 °C. In sharp contrast, for Co/SiC the Co-based specific productivity was only 35 g_H_2__ g_Co_^−1^ h^−1^ at 550 °C and increased up to 111 g_H_2__ g_Co_^−1^ h^−1^ at 582 °C. Remarkably, the SCALMS productivity outperformed the monometallic Co/SiC by a factor of 14 at a temperature of 550 °C. We hypothesize that this drastic increase in activity is related to the single-atom nature of Co at the catalytic interface of the SCALMS system and due to the high mobility of the supported liquid alloy at this elevated temperature.^22–24^ The mobility of these Co single atoms may help to recombine adsorbed nitrogen atoms to form N_2_ which is the commonly reported rate determining steps in ammonia decomposition for metals like Co and Ni.^9,10,47^ Additionally, there is a possibility of a spillover of nitrogen to Ga, accompanied by the formation of dynamic Ga–N species, as reported by Zuraiqi *et al.* during ammonia synthesis utilizing liquid metal Ga–Cu.^21^ Analogous effects for Ga–Rh SCALMS in the activation of propane during propane dehydrogenation have been reported by our group.^11^ Furthermore, the specific electronic nature of Co surrounded by Ga atoms at the liquid metal interface is expected to further promote nitrogen recombination by an electron donation effect.^11,43,48–52^

**Fig. 4:**
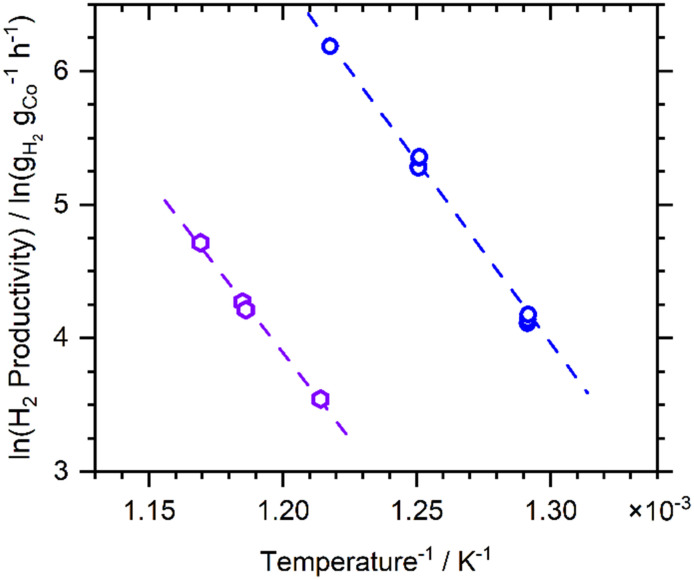
Arrhenius plot using the natural logarithm of the Co mass-specific H_2_ productivity at 5 ± 0.5% NH_3_ conversion plotted over the inverse catalyst temperature for Ga_59_Co/SiC (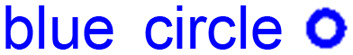, *m*_cat_ = 1.00 g, *V*_cat_ = 1.8 ml, *w*_Co_ = 0.04 wt%, *w*_Ga_ = 3.12 wt%, WHSV = 4–29 l_N_ g_cat_^−1^ h^−1^, *p*_total_ = 1 bar(a)) and Co/SiC (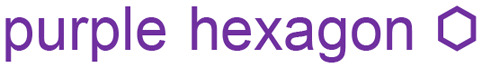, *m*_cat_ = 1.00 g, *V*_cat_ = 1.5 ml, *w*_Co_ = 0.05 wt%, WHSV = 3–9 l_N_ g_cat_^−1^ h^−1^, *p*_total_ = 1 bar(a). Dashed lines indicate linear fits.

Fig. 4 shows the Arrhenius plots using the natural logarithm of the H_2_ productivities for both catalysts. Equal conversion rates were used to avoid influences of different product partial pressures and of different distances from equilibrium conversion. The slope of the plotted data was used to calculate the apparent activation energies (*E*_A,app_) for both systems. They were determined to be 226 ± 6 kJ mol^−1^ for Ga_59_Co/SiC SCALMS and 215 ± 9 kJ mol^−1^ for the monometallic Co/SiC, respectively. From these data, we conclude that both reactions take place in the kinetic regime.^53^ This is no surprise given the meso- and microporous nature of the applied SiC support and the fact that our SCALMS preparation by ultrasonication leads to support decoration with alloy droplets mainly on the outer surface (see SI for the respective microscopic results, Fig. S8).^12,16,41,42^ Film diffusion limitation can be safely excluded from the *E*_A,app_ values determined.^53^

Co-catalyzed ammonia decomposition is reported to be a structure sensitive reaction. Supported Co particle in the size range of 10 to 20 nm have been shown to exhibit the highest activities.^37,52,54–57^ Therefore, we prepared a Co catalyst on SiC with a 21 times higher Co loading (1.05 wt%) leading to particle sizes of about 10 nm as confirmed by STEM–EDX images (see Fig. S6). The experiments with this higher loaded and ideally sized Co/SiC catalyst showed indeed 1.6 higher activity (comparison at 550 °C), but still the SCALMS catalyst remains 9 times more active than this optimized Co/SiC material (see Fig. S11). Table 2 compares the hydrogen productivities obtained in this study to other reported Co-based ammonia decomposition catalysts from the literature. Such comparison is challenging, as also addressed by Ristig *et al.*,^9^ because of the variability of conditions and conversion levels as well as sometimes lack of detailed experimental information in the ammonia decomposition literature. Despite these difficulties, it is fair to state that the hydrogen productivity of our Ga_59_Co/SiC SCALMS catalyst exceeds all reported example of Co-based ammonia decomposition catalysts at 500 and 550 °C by far. Note that the productivity of our monometallic Co/SiC catalyst is well in line with other monometallic Co systems described in the literature and shown in Table 2. This underscores the considerable potential of SCALMS-type catalysts for the further development of ammonia decomposition towards productivity levels that were previously unattainable for base metals and reserved for the use of precious metals, such as Ru-based catalysts.^9,58–60^

**Table 2:** Co mass-based hydrogen productivity through ammonia decomposition of various SiC supported Co-based catalyst – comparison of data from this work with literature data obtained under comparable conditions

Catalyst	Co loading/wt%	WHSV/ml_N,NH_3__ g_cat_^−1^ h^−1^	Temperature/°C	NH_3_ conversion/%	H_2_ productivity/g_H_2__ g_Co_^−1^ h^−1^	Ref.
Ga_59_Co/SiC	0.04	29 000	550	5.1	487	This work
Co/SiC	0.05	3000	550	4.6	34	This work
Co/SiC	1.05	91 200	550	4.6	54	This work
Co/SiC	10	30 000	550	43.8^a^	18^b^	61
Co/SiC	25	30 000	550	73.2	12^b^	61
Co/SiC	35	30 000	550	78.3	12^b^	62
Co/SiO_2_	10	30 000	550	70	28	57, 63
5CoNa/Ti-NT	3.5	6000	550	23	5	56
Co/CNTs	5.0	36 000	550	—	29^b^	64
Ga_59_Co/SiC	0.04	4500	500	4.8	65	This work
Co/SiC	1.05	18 700	515	4.7	12	This work
Co/SiO_2_	10	30 000	500	42.2	17	63
Co/BaNH	4.8	36 000	500	—	50^b^	64
Co/CNTs	5.0	36 000	500	—	13^b^	64
Co/CNTs	4.1	5000	500	8	1.5	57, 65
Co in Al_2_O_3_ matrix	95	18 000	500	72	9	52, 66

a Determined from graphs using the open source software ImageJ.^67^

b Calculated from published and determined data.

## Conclusion

In summary, we have demonstrated in this work the substantial potential of Ga–Co SCALMS materials as heterogeneous catalysts for the decomposition of ammonia under technical relevant conditions. The SCALMS concept appears to be highly beneficial for specific requirements of ammonia decomposition: i) it offers a very efficient use of Co due to the single atom nature of Co in Ga-based SCALMS; ii) the Ga surrounding of Co seem to alter the electronic properties of Co in a beneficial way for ammonia decomposition; iii) the highly dynamic liquid alloy nature seem to promote the recombination of adsorbed nitrogen atoms to form N_2_. These beneficial effects and their combination open new avenues for the further optimization of ammonia decomposition catalysis, avenues that go clearly beyond traditional structure-sensitive surface catalysis and avoid the use of precious metals.

## Author contributions

Philipp Rothgängel: resources, investigation, formal analysis, validation, visualization, writing – original draft preparation and data curation. Nicola Taccardi: resources and methodology (SCALMS catalysts), writing – review & editing. Aaron Luke Folkard: resources and methodology. Jakob Söllner: investigation, formal analysis and visualization. Alexander Søgaard: resources. Andreas Körner: investigation and visualization. Andreas Hutzler: supervision and investigation. Matthias Thommes: supervision and funding acquisition. Marco Haumann: writing – review & editing, conceptualization, supervision, project administration, funding acquisition. Peter Wasserscheid: writing – review & editing, conceptualization, supervision, project administration, funding acquisition. All authors have given approval to the final version of the manuscript.

## Conflicts of interest

There are no conflicts to declare.

## Supplementary Material

CY-016-D6CY00362A-s001

## Data Availability

Data for this article, including figures and plot data are available at Zenodo at https://doi.org/10.5281/zenodo.14968866. The data supporting this article have been included as part of the supplementary information (SI). Supplementary information is available. See DOI: https://doi.org/10.1039/d6cy00362a.
