# Supplementary material for: Ambient pressure ammonia decomposition using Ga–Co supported catalytically active liquid metal solutions
Source: Catal Sci Technol. 2026 Jun 6;16(12):4019–27. doi: 10.1039/d6cy00362a (PMC13250925; doi:10.1039/d6cy00362a)
Supplement: CY-016-D6CY00362A-s001 [file CY-016-D6CY00362A-s001.pdf]

Supplementary Information (SI) for Catalysis Science & Technology.
This journal is © The Royal Society of Chemistry 2026

Supporting Information

Ambient Pressure Ammonia Decomposition Using Ga-Co Sup-ported Catalytically Active Liquid Metal Solutions

Philipp Rothgängel [a b], Nicola Taccardi [c *], Aaron Luke Folkard [c d], Jakob Söllner [c], Alexander Søgaard [c], Andreas Körner [a], Andreas Hutzler [a], Matthias Thommes [e], Marco Haumann [c], Peter Wasserscheid [a b c *]

[a] Forschungszentrum Jülich GmbH, Helmholtz-Institute Erlangen-Nürnberg for Renewable Energy (IET-2), Cauerstraße 1, Erlangen 91058, Germany

[b] Forschungszentrum Jülich GmbH, Institute for a sustainable Hydrogen Economy (IHE), Am Brainergy Park 4, 52428, Jülich, Germany

[c] Friedrich-Alexander-Universität Erlangen-Nürnberg (FAU), Lehrstuhl für Chemische Reaktionstechnik (CRT), Egerlandstr. 3, Erlangen 91058, Germany

[d] University of KwaZulu-Natal, School of Chemistry and Physics, Durban, KwaZulu Natal, South Africa

[e] Friedrich-Alexander-Universität Erlangen-Nürnberg (FAU), Lehrstuhl für Thermische Verfahrenstechnik (TVT), Egerlandstr. 3, Erlangen 91058, Germany

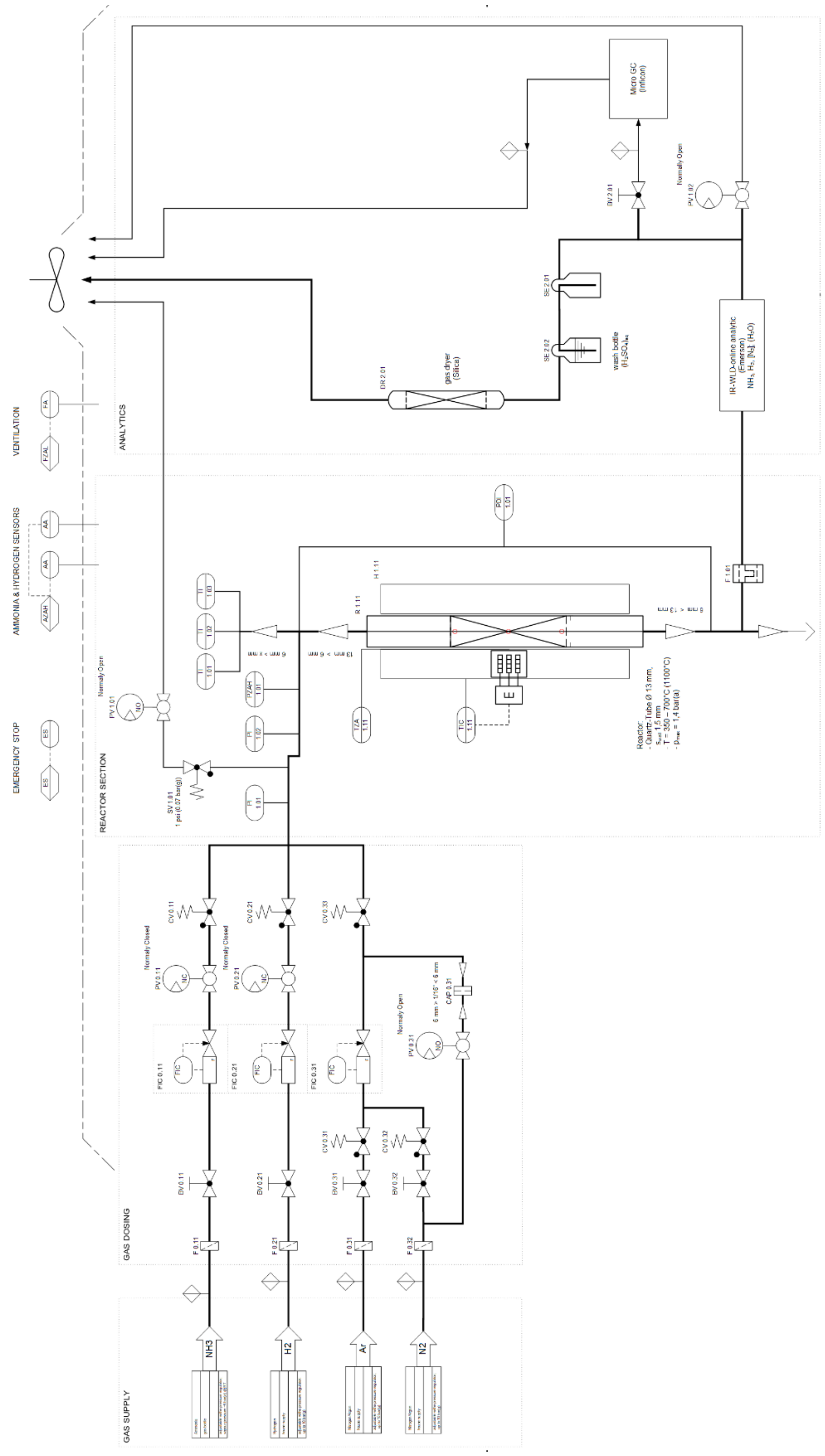

Continuous gas-phase reactor setup

A detailed flow scheme of the reactor is shown in Figure S1.

Figure S 1. R&I of the continuous gas-phase reactor used for ammonia decomposition studies.

Calculations and Equations

The weight hourly space velocity (WHSV) is defined as the ratio of the total gas flow, in our case the volume flow of pure NH3, at standard conditions (German: Normbedingungen) 273.15 K and 1013.25 mbar ($\dot{V}_N$) over the catalyst mass (m_{cat}).

$$\mathrm{WHSV} = \frac{\dot{V}_N}{m_{cat}} \tag{S 1}$$

The gas hourly space velocity (GHSV) is defined as the ratio of the total gas flow, in our case the volume flow of pure NH_3, at standard conditions (German: Normbedingungen) 273.15 K and 1013.25 mbar ($\dot{V}_N$) over the catalyst bed volume (V_{cat}).

$$\mathrm{GHSV} = \frac{\dot{V}_N}{V_{cat}} \tag{S 2}$$

The catalyst bed volume V_{cat} was determined by measuring the height of the catalyst bed (h_{cat}) with a string spacing ruler (accuracy ± 0.5 mm), the inner diameter of the reactor ($d_{in,reactor}$) and the outer diameter of the inner capillary ($d_{out,capillary}$) according to the following equation:

$$V_{cat} = \frac{\pi}{4}\left(d_{in,reactor}^2 - d_{out,capillary}^2\right)\cdot h_{cat} \tag{S 3}$$

The ammonia conversion (X_{NH3}) was calculated using the normalized molar concentrations of NH_3 (x_{NH3}) obtained from the micro gas chromatograph.

$$X_{NH_3} = \frac{1 - x_{NH_3}}{1 + x_{NH_3}} \tag{S 4}$$

The cobalt specific H_2 productivity was defined as the released H_2 (product of $\mathbf{X_{NH3}}$ with the molar ratio 1.5 between NH_3 and H_2 according to the reaction equation) multiplied with the molar flow of ammonia (product of $\mathbf{\dot{V}_N}$, NH_3 density at standard condition $\boldsymbol{\rho_{NH3}}$, through molar mass $\mathbf{M_{NH3}}$) and divided by the utilized Co mass $\mathbf{m_{Co}}$ (product $\mathbf{m_{cat}}$ and Co mass loading $\mathbf{w_{Co}}$):

$$\text{specific } H_2 \text{ productivity} = 1.5 \cdot X_{NH_3} \cdot \frac{\dot{V}_N \cdot \rho_{NH_3}}{M_{NH_3}} \cdot \frac{1}{m_{cat} \cdot w_{co}} \tag{S 5}$$

Analogously, H_2 productivities specific to other metals were calculated.

Materials

Material / product name	CAS nr.	Batch nr.	Purity	Supplier
β-SiC / SiC3-E2-HP	409-21-2	SD0222C3W	> 99.9%	ACM GmbH
β-SiC / SiC4-E2-HP	409-21-2	SD0222A3W	> 99.9%	ACM GmbH
Ga	7440-55-3		99.9999%	ThermoScientific
$Co(NO_3)_2 \cdot 6H_2O$ / Cobalt(II) nitrate hexahydrate	10026-22-9	M16D012	98-102%	Alfa Aesar
$Ni(DME)Cl_2$ / nickel(II) chloride ethylene glycol dimethyl ether	29046-78-4		98%	Sigma-Aldrich
$CuCl_2$ / copper(II) chloride	7447-39-4		≥ 99.995%	Sigma-Aldrich
Propan-2-ol / isopropanol	67-63-0		≥99.3%	Jäkle Chemie (Germany)
NH_3 / Ammonia 5.0	7664-41-7		≥ 99,999%	Air Liquide Deutschland GmbH
H_2 / ALPHAGAZ™1 Hydrogen (5.0)	1333-74-0		≥ 99,999 mol%	Air Liquide Deutschland GmbH
N_2 / ALPHAGAZ™1 Nitrogen (5.0)	7727-37-9		≥ 99,999 mol%	Air Liquide Deutschland GmbH
Ar / ALPHAGAZ™1 Argon (5.0)	7440-37-1		≥ 99,999 mol%	Air Liquide Deutschland GmbH

Materials Synthesis

Method for Monometallic Co on SiC

Incipient wetness impregnation was used to prepare monometallic Co on SiC catalysts.

Aiming for 0.07 wt% (ICP-AES: 0.05 wt%): 0.0518 g of $Co(NO_3)_2 \cdot 6H_2O$ was weighed and dissolved in 30 mL of de-ionized water. The Co solution was slowly added dropwise to 15.0052 g of SiC4 (250-500 µm), until the support was slightly wet.

Aiming for 1 wt% Co loading (ICP-AES: 1.05 wt%): 0.3965 g of $Co(NO_3)_2 \cdot 6H_2O$ was weighed and dissolved in 15 mL of de-ionized water. The Co solution was slowly added dropwise to 7.9170 g of SiC4 (250-500 µm), until the support was slightly wet.

The mixture was then rotated at 250 rpm for 10 min before drying at 100 °C for 30 min.

The resultant Co-SiC sample was then calcined using the following procedure:
The sample was heated from room temperature to 150 °C (at a rate of 2 °C min^{-1}) and then held at 150 °C for 30 min; thereafter heated from 150 °C to 600 °C (at a rate of 2 °C min^{-1}) and then held at 600 °C for 180 min prior to returning to room temperature.

Method for SCALMS catalyst

The SCALMS catalysts were prepared by physical dispersion of the matrix metal Ga, followed by the deposition of the dispersion on SiC3 support.

The first step includes the deposition of Ga on the support. A Ga nugget of about 1.00 g was melted and dispersed in 100 mL of isopropanol via ultrasonication. Ultrasonication was carried out at 40 °C using Branson 450D sonicator (400 W) equipped with a ¼” tip at 80% amplitude (power input 80 W) for 30 min. After sonication, the emulsion was added to a defined amount of SiC (~13.00 g). Then, the solvent was slowly evaporated in a rotary evaporator at 50 °C. The obtained solid was calcined at 450 °C for 2 h.

For the second step of the preparation procedure the resulting Ga/SiC was suspended in 50 mL of isopropanol and solution of the corresponding amounts of metal precursors, namely Cobalt(II) nitrate hexahydrate, nickel(II) chloride ethylene glycol dimethyl ether, and copper(II) chloride, dissolved in 10 mL $20_{v/v}$% water in isopropanol, was added in order to achieve the desired Ga/secondary metal ratio. The solvent was then slowly evaporated in a rotary evaporator at 50 °C. The obtained solid was calcined at 500 °C for 2 h.

Mercury Intrusion Porosimetry

Mercury intrusion porosimetry was performed on a Poremaster60 (Anton Paar QuantaTec, FL, USA). Triple distilled mercury (purity 99.9995%) was purchased from GMR Gesellschaft für Metallrecycling GmbH, Leipzig. All measurements were performed using the Autospeed mode with motor speed 1. Outgassing of the samples was done at ambient temperature using the vacuum procedure in the experimental procedure. Data evaluation was done using the corresponding software (PoroWin, Anton Paar Quantatech, Fl. USA) utilizing the Washburn equation with 480 mN m^{-1} as the liquid vapor surface tension of mercury and 140° as the contact angle in between mercury and SiC. Figure S 3 shows the cumulative intrusion curves including the first intrusion step, associated with powder/particle compression. The pore size distributions are shown in Figure S 2.

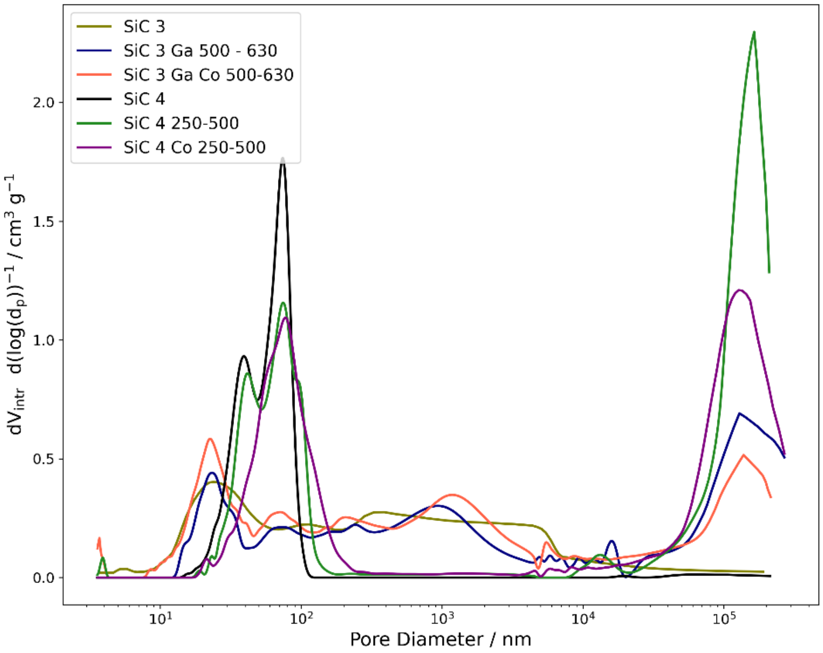

Figure S 2: Differential pore size distributions for SiC support (SiC3 whole pellets, SiC4 crushed pellets sieve fractions 250 - 500 µm and 500 - 630 µm), $Ga_{59}Co$/SiC3 (crushed pellets sieve fractions 500 - 630 µm), 1.05 wt% Co/SiC4 (crushed pellets sieve fraction 250 - 500 µm) and Ga/SiC3 (crushed pellets sieve fractions 500 - 630 µm)

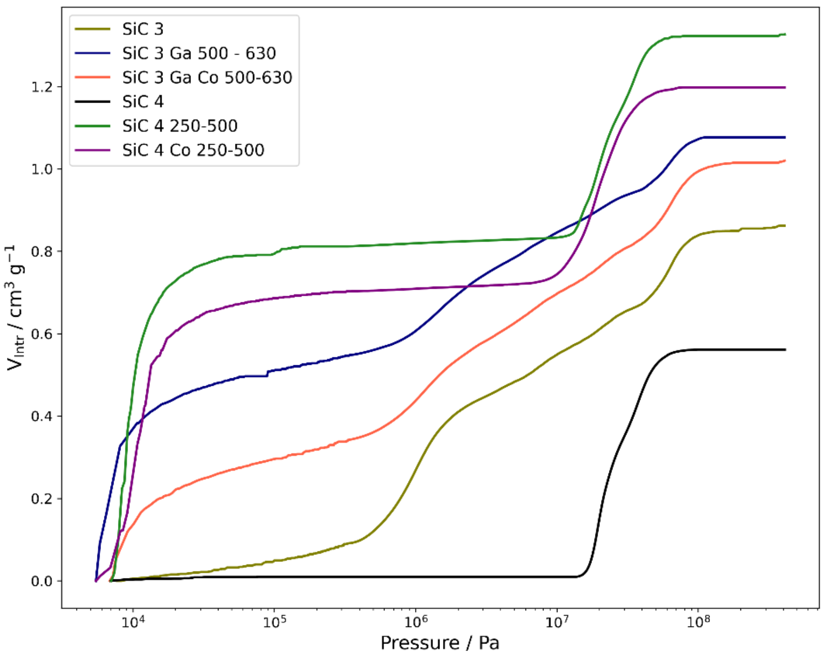

Figure S 3: Cumulative mercury intrusion volume over applied total pressure for SiC support (SiC3 whole pellets, SiC4 crushed pellets sieve fractions 250 - 500 µm and 500 - 630 µm), Ga59Co/SiC3 (crushed pellets sieve fractions 500 - 630 µm), Co/SiC4 (crushed pellets sieve fraction 250 - 500 µm) and Ga/SiC3 (crushed pellets sieve fractions 500 - 630 µm)

Nitrogen Sorption

The N_2 sorption full isotherms (see Figure S 4 and Figure S 5) were measured with a TriStar II Plus (Micromeritics GmbH) after predrying the samples at 573 K for at least 12 h. Data acquisition, calculations and model fitting ware done with the corresponding manufacturer software TriStar II Plus version 3.02. Data analysis based on the multipoint BET model were used to obtain information about the specific surface area and are shown in Table S 1.

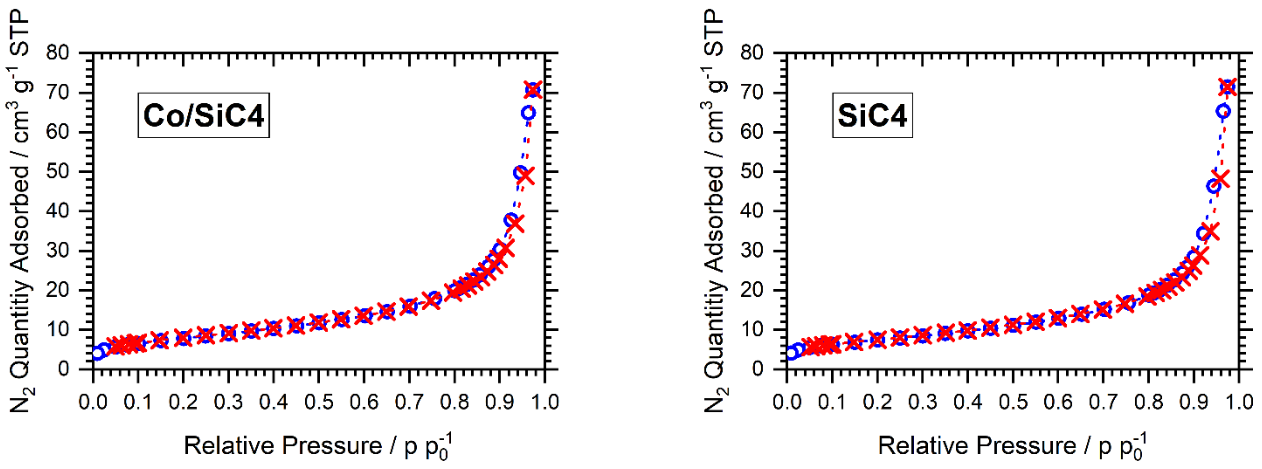

Figure S 4: Adsorption (red cross x) and desorption (blue open circle ○) isotherms for 1.05 wt% Co on SiC4 (sieve fraction 250 – 500 µm, left diagram) before reaction in comparision with the untreated raw SiC4 (sieve fraction 250 – 500 µm, right diagram). Analysis adsorptive N_2, analysis bath temperature 77.3 K, saturation pressure p_0 993 mbar (Co/SiC4) and 992 mbar (SiC3).

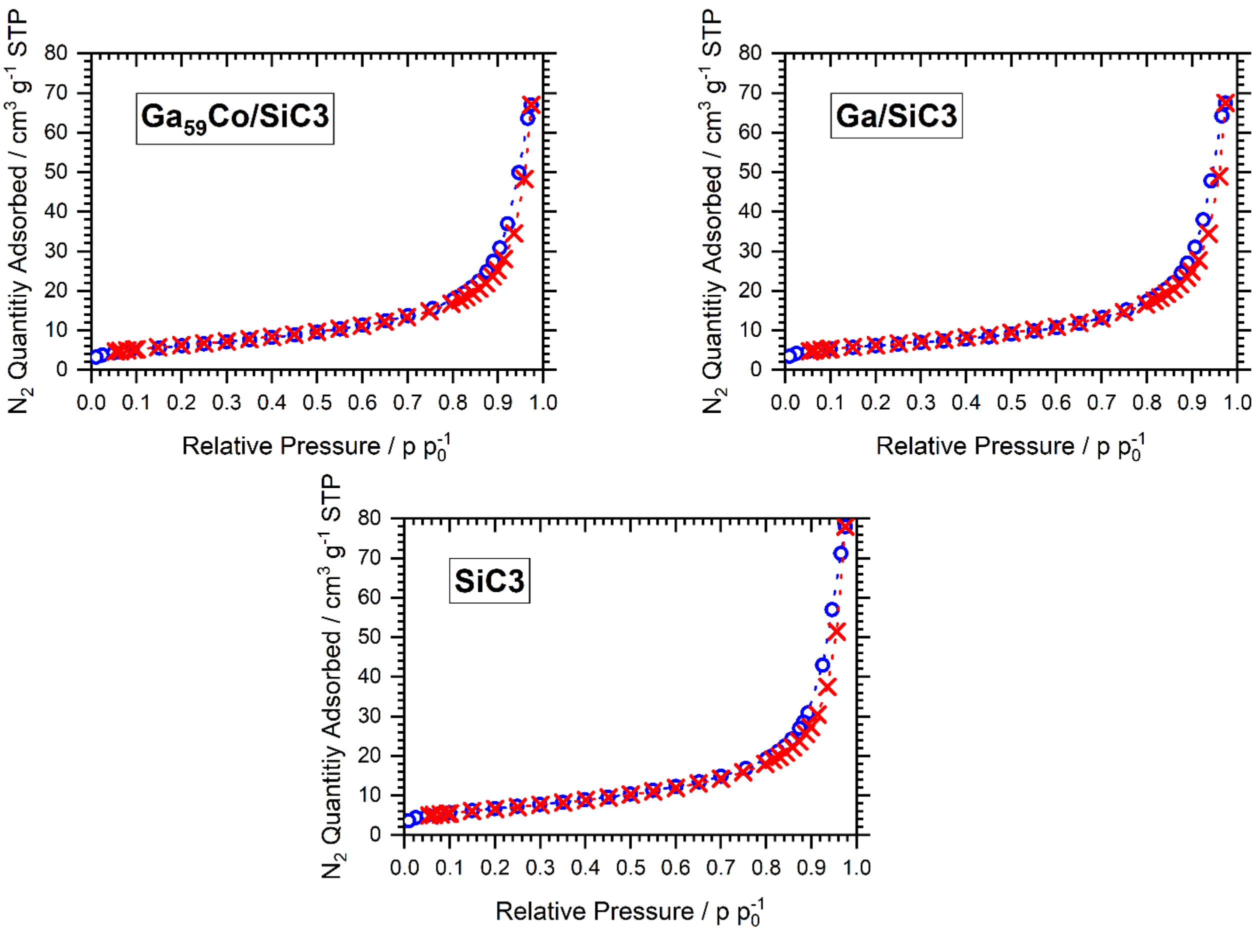

Figure S 5: Adsorption (red cross x) and desorption (blue open circle ○) isotherms for $Ga_{59}Co$ on SiC3 (sieve fraction 500 – 630 µm, upper left diagram) and Ga on SiC3 (sieve fraction 500 – 630 µm, upper right diagram) before reaction in comparision with the untreated raw SiC3 pellets support. Analysis adsorptive N_2, analysis bath temperature 77.3 K, saturation pressure p_0 991 mbar ($Ga_{59}Co$/SiC3), 992 mbar (Ga/SiC3) and 1000 mbar (SiC3).

Table S 1: Material properties retrieved by N_2 sorption and Hg porosimetry.

Material	Particle size* / µm	Surface area** / $m^2\ g^{-1}$	Hg porosimetry*** / $cm^3\ g_{support}^{-1}$			
			5 – 50 nm	< 5 µm	> 10 µm	Total intrusion volume
SiC3	-	24.01 $^{\pm 0.04}$	0.21	0.78	0.06	0.86
$Ga_{59}Co$/SiC3	500 - 630	22.87 $^{\pm 0.02}$	0.14	0.53	0.52	1.08
Ga/SiC3	500 - 630	22.54 $^{\pm 0.04}$	0.21	0.68	0.31	1.02
SiC4	-		0.22	0.55	0.01	0.56
SiC4	250 - 500	27.16 $^{\pm 0.02}$	0.17	0.51	0.81	1.33
Co/SiC4	250 - 500	29.29 $^{\pm 0.03}$	0.09	0.50	0.69	1.20

*sieve fraction after crushing pellets **by multipoint BET ***separated by intra-particle pore diameters

Scanning Transmission Electron Microscopy – Energy Dispersive X-Ray Spectroscopy (STEM -EDXS)

A Talos F200i from Thermo Fisher Scientific was used to record images with a high angle annular dark field (HAADF) STEM detector. The microscope is equipped with a Schottky emitter, two Bruker XFlash 6T-100EDS detectors and was operated at 200 kV acceleration voltage. Data evaluation was performed in the software Velox.
The samples were prepared by grinding the catalyst in a mortar to receive a fine powder, which was dispersed in a mixture of ethanol and isopropanol 50:50 (volume) within a rolled rim glass and sonicated for at least 5 min. The dispersion was drop-casted on a TEM grid placed on a clean filter paper and dried in air on ambient conditions. The TEM-grids used were distributed by Plano GmbH and manufactured by Quantifoil Micro Tools GmbH as a gold grid (mesh size 300) with a holey carbon film. The measurements and images for samples with 0.05 wt% Co/SiC4 are not shown, as no Co was detectable in the samples before or after the reaction due to the low metal loading. Therefore, Co signals get lost in the background noice.
As the smallest clusters we could distinguish in the 1.05 wt% Co/SiC4 samples (Figure S 6) were about 5 nm in diameter, we can expect the Co in the 0.05 wt% samples to be evenly distributed in SiC or to be in clusters significantly smaller than 5 nm.

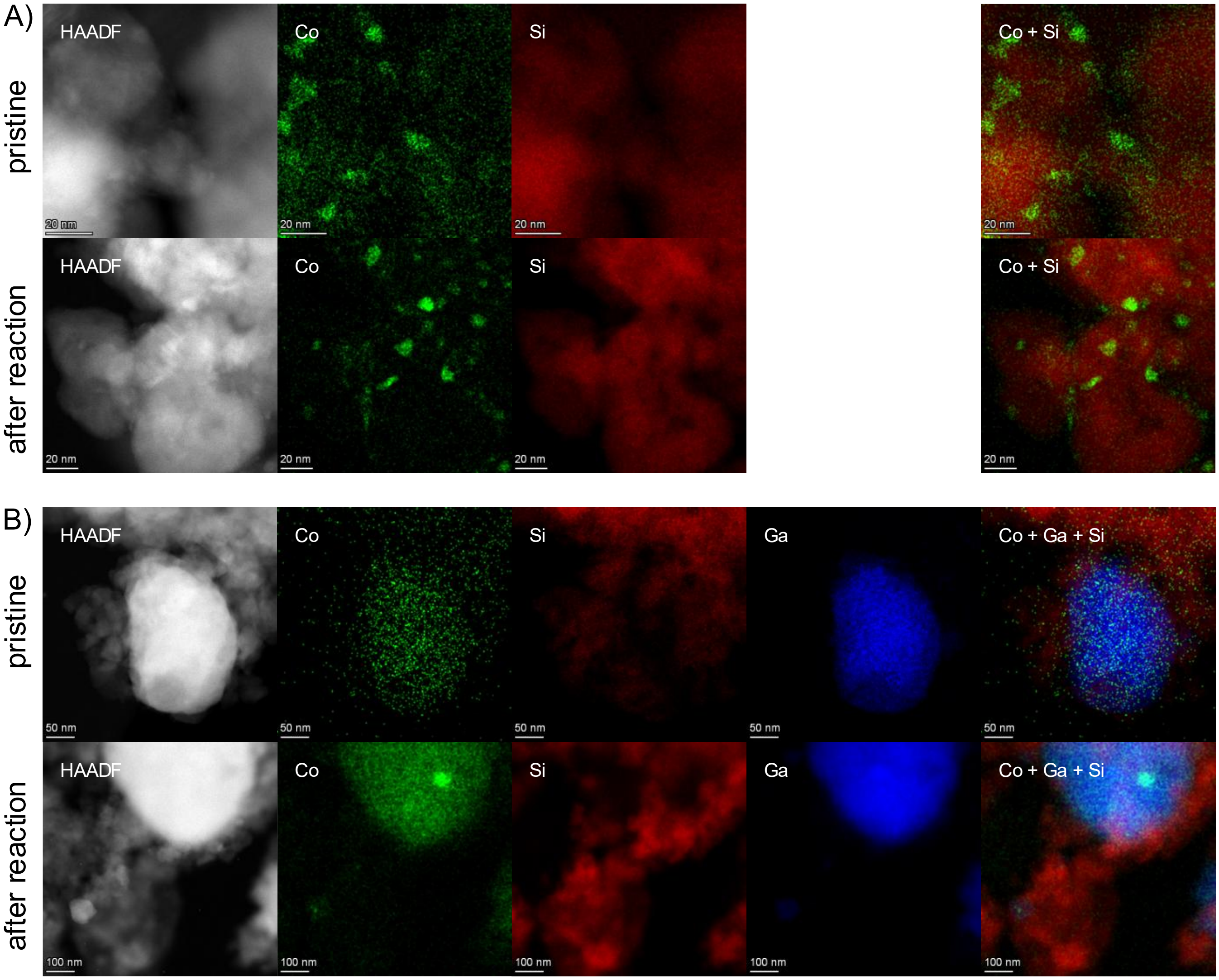

Figure S 6: STEM-EDX spectrum imaging of A) Co/SiC4 catalyst containing 1.05 wt% Co only. Individual particles are easily identified both in the pristine sample and after cycling. B) $Ga_{59}Co/SiC3$ catalyst containing Ga and Co. Note that Co here is always associated with Ga.

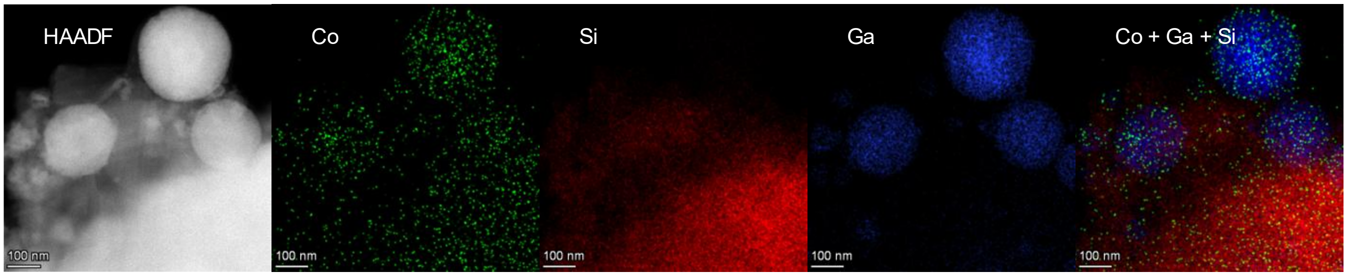

Figure S 7: STEM-EDX spectrum imaging of $Ga_{59}Co/SiC3$ catalyst after the reaction containing Ga and Co. Here the Ga-droplets were exfoliated with adhesive tape to isolate Ga-Co droplets from the structure. The tape used was a conductive double sided adhesive carbon tape (MSPP15-000512) supplied by VWR International GmbH. Note that again Co here is always associated with Ga and no isolated Co particles were observed.

Scanning Electron Microscopy - Energy Dispersive X-Ray Spectroscopy (SEM-EDXS)

Scanning Electron Microscopy (SEM) analyses were carried out using a Phenom Desktop SEM (full backscattered electron detector (BSD), 15 kV voltage). The particles were deposited directly on a conductive sticky carbon tape (MSPP15-000512) supplied by VWR International GmbH and dried under ambient conditions before measurement. Figure S 8 shows an exemplary SiC3 particle coated with $Ga_{59}Co$ droplets. The droplets enter pores and cavities at the surface with diameters of 10 µm and above.

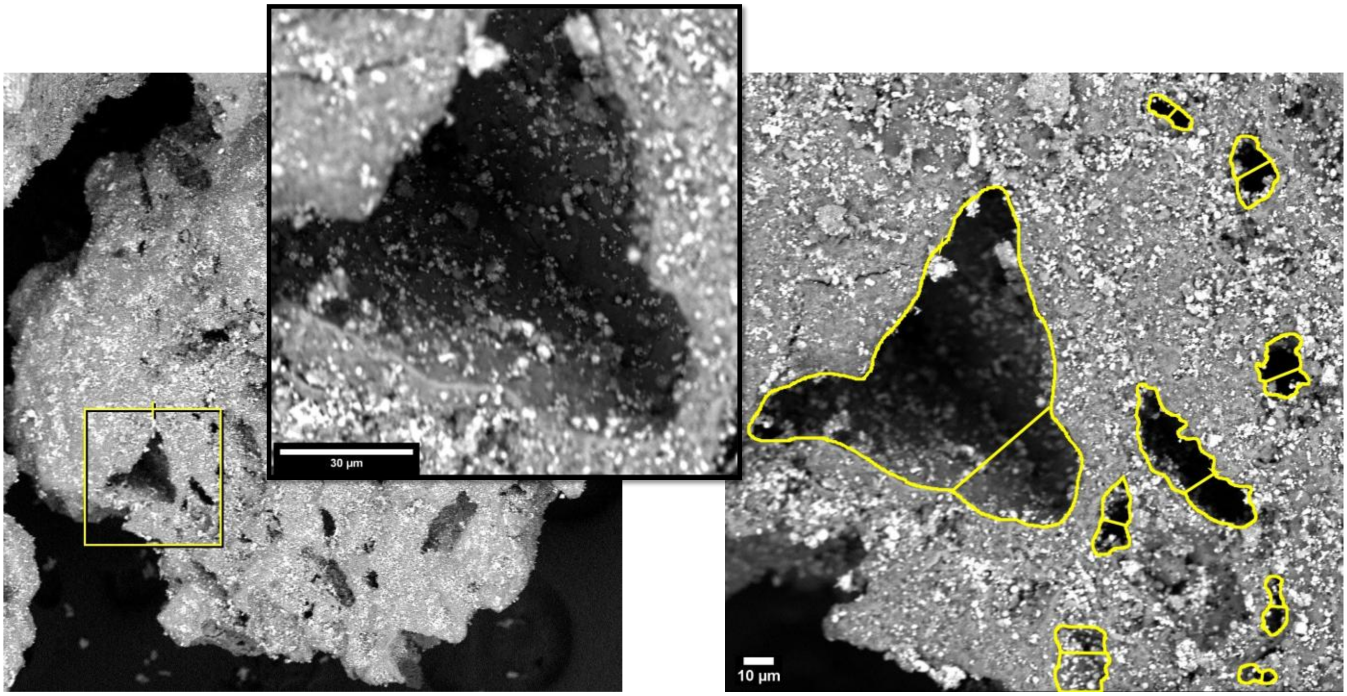

Figure S 8: Exemplary SEM images (BSD Full) of an $Ga_{59}Co/SiC3$ catalyst with highlighted pores and cavities containing metal droplets.

Inductively Coupled Plasma Atomic Emission Spectroscopy (ICP-AES)

The Ga and Co loading and the corresponding Ga:Co-ratios of the SCALMS were determined by inductively coupled plasma atomic emission spectroscopy (ICP-AES) using a Ciros CCD (Spectro Analytical Instruments GmbH). The same was done for the Ni and Cu. The solid samples were ground with a mortar prior to digestion in solution of concentrated HCl:HNO_3:HF (volumetric ratio of 3:1:1) using microwave heating up to 220 °C for 40 min.
The instrument was calibrated with standard solutions of Co, Ni, Cu and Ga prior to the measurements.

X-Ray diffraction (XRD)

Powder X-ray diffraction (XRD) measurements were performed on a X'Pert Pro by Malven Panalytics to determine the solid-state structure of the prepared, mortar grounded materials. The measurements were carried out in angles 2θ of 10–90 °, a scanning speed of 0.02°/s, a step size of 0.015° and 100 s time per step. Cu−Kα was used as the radiation source and X'Celerator as the detector. The data was processed utilizing X'Pert Highscore Plus and compared to simulated reflexes from the inorganic crystal structure database (ICSD).

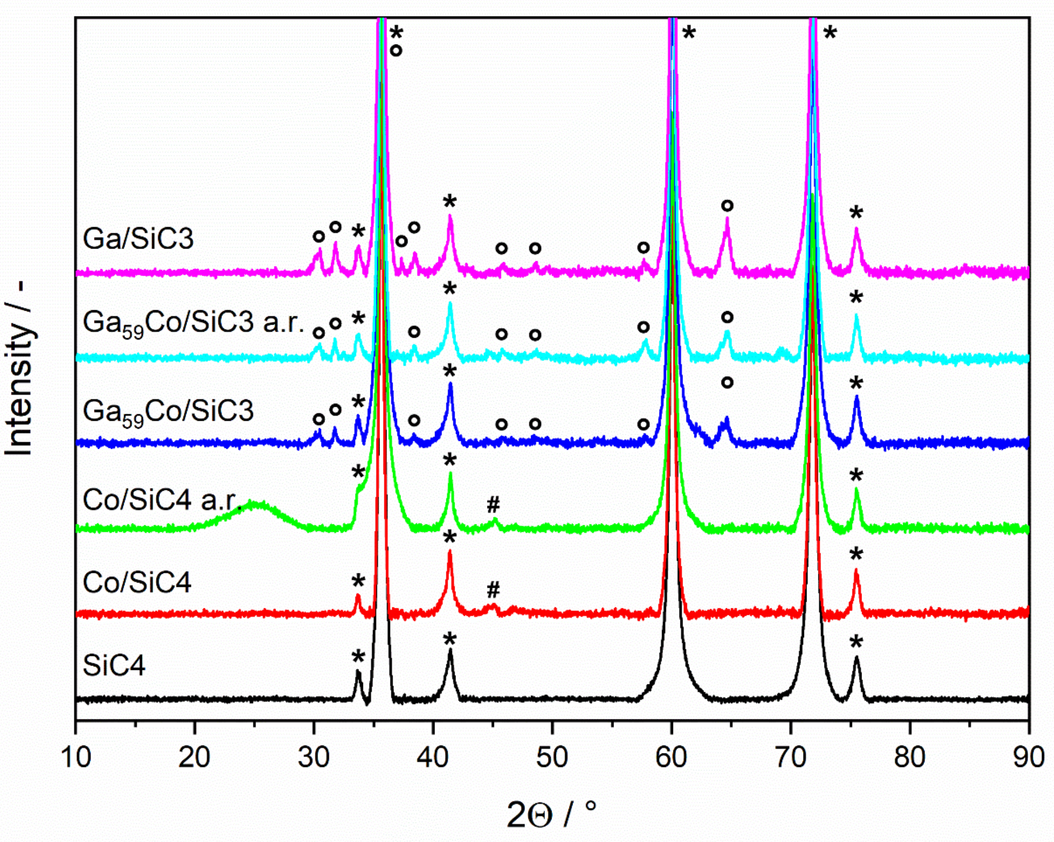

Figure S 9: XRD patterns of the samples (bottom to the top) SiC4 (black), 1.05 wt% Co on SiC4 before (red) and after reaction (green), $Ga_{59}Co$ on SiC3 before (dark blue) and after reaction (cyan) and Ga on SiC (pink). The symbols refer to dominant reflexes for SiC/Moissanite 3C (star: *****, ICSD-reference code 98-016-4973), gallium oxide β (circle °, ICSD-reference code 98-003-4243). The weak reflex at close to 45° observed for monometallic Co on SiC systems (hashtag: **#**) could refer to dicobalt silicide (ICSD-reference code 98-005-2281), but could not be clarified due to the low reflex intensity.

The XRD data give no hint for a Ga-Co intermetallic structure, but as the Co related reflex for the monometallic Co on SiC system with 1.05 wt% Co loading 2θ at about 45° was low in intensity, Co related reflexes for the SCALMS system with 0.04 wt% Co loading are expected to be not measurable in this setup. Therefore, the measurement for 0.05 wt% Co on SiC was skipped. No shifts in the Ga related reflexes for the $Ga_{59}Co$/SiC3 were observable.

Blank activity quartz reactor and supporting materials

A systematic approach was chosen to verify the blank activity of the quartz reactor tube, quartz capillary, quartz wool, activated carbon and SiC support, as well as to check the blank activity of pure gallium. The quartz reactor was loaded with 0.3 g of quartz wool, 0.3 g of activated carbon, and 1.05 g of SiC support, loaded with 4.27 wt% Ga. The system was reduced under a gas flow of a 3:1 H_2:N_2 mixture at up to 640 °C. The gas was switched to an undiluted NH_3 gas flow in the range of 190-60 ml_N min^{-1} and the temperature was stepwise increased up to 582 °C (see Figure S 10).

Within the first 30 min, an apparent conversion, decreasing over time, was observable and is accounted to remnants of the reduction gas being flushed out. Up to temperatures of 582 °C and flowrates down to 60 ml_N min^{-1} (equals a WHSV of 3 l_N g_{cat}^{-1} h^{-1}) stayed below the threshold value of 0.05%. No N_2 was detectable after 0.5 h TOS during the experiment. H_2 signals were negligible low. Therefore, the combined activities of the Ga on SiC, quartz components, and activated carbon are negligible within the parameter ranges of the presented experiments. The blank activities of the isolated components are consequently also considered negligible, and no influence on the accuracy of the experiments is anticipated.

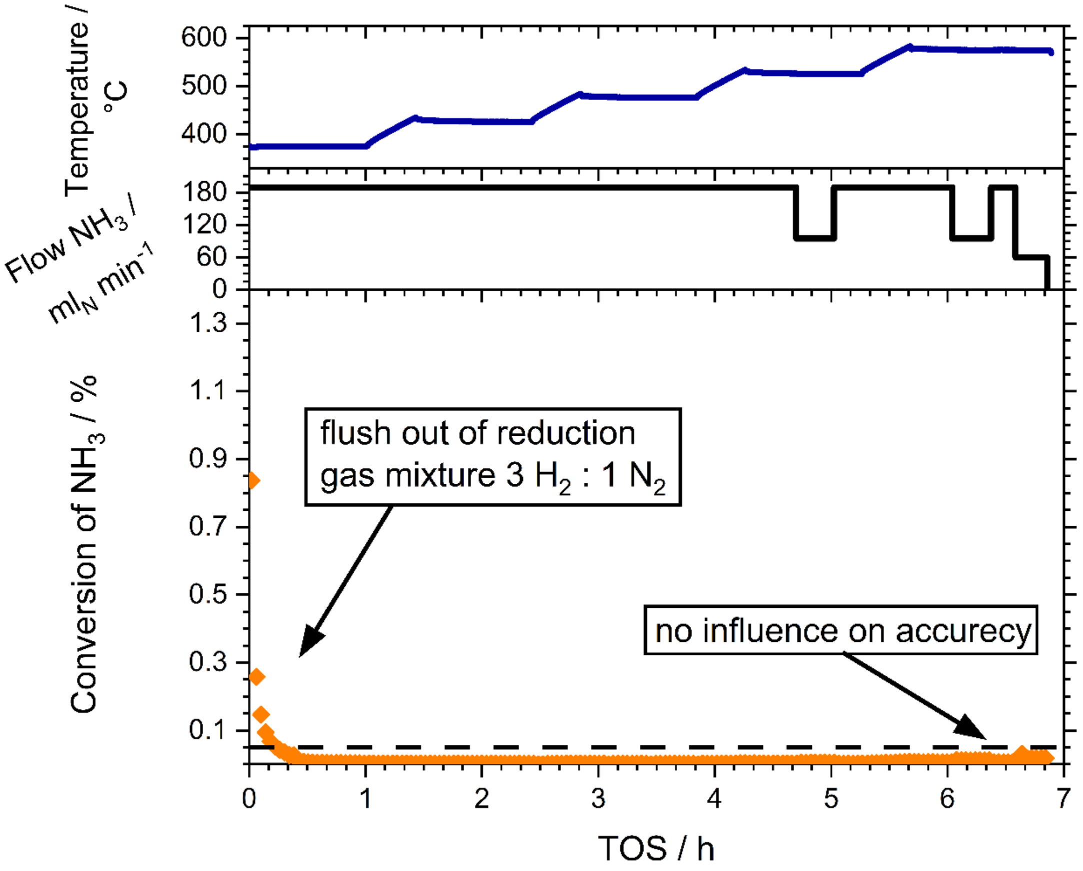

Figure S 10: Catalyst temperature (top; **blue line**), NH_3 flowrate (middle; **black line**), and NH_3 conversion for Ga on SiC as blank conversion test and as reference (bottom, **orange diamond** ◆, m_{cat} = 1.05 g, V_{cat} = 2.2 ml, w_{Ga} = 4.27 wt%, WHSV = 3-11 l_N g_{cat}^{-1} h^{-1}, p_{total} = 1 bar(a), $T_{cat,max}$ = 582 °C). The dashed line indicates the 0.05% conversion threshold below which no influence on the accuracy of the experiment is claimed.

Extended Co-specific H_2 productivity data

A comparison of Co-specific H_2 productivity over temperature for a $Ga_{59}Co/SiC3$ SCALMS (0.04 wt% Co-loading) with two monometallic Co/SiC4 catalysts (1.05 wt% and 0.05 wt% Co-loading) is given in Figure S 11. Noteworthy is the up to 9 times higher productivity of the SCALMS catalyst at comprising temperatures of 550 °C and 527 °C with 487 to 54 $g_{H2}\ g_{Co}^{-1}\ h^{-1}$ and 198 to 21 $g_{H2}\ g_{Co}^{-1}\ h^{-1}$ respectively. Between the two monometallic Co catalysts at 550°C a factor of 1.6 in the Co specific productivity was observable, with the higher loaded catalyst (1.05 wt%) being more active. This observation can be explained by the bigger Co clusters in the 1.05 wt% Co/SiC4 confirmed in the STEM-EDXS images (Figure S 6).

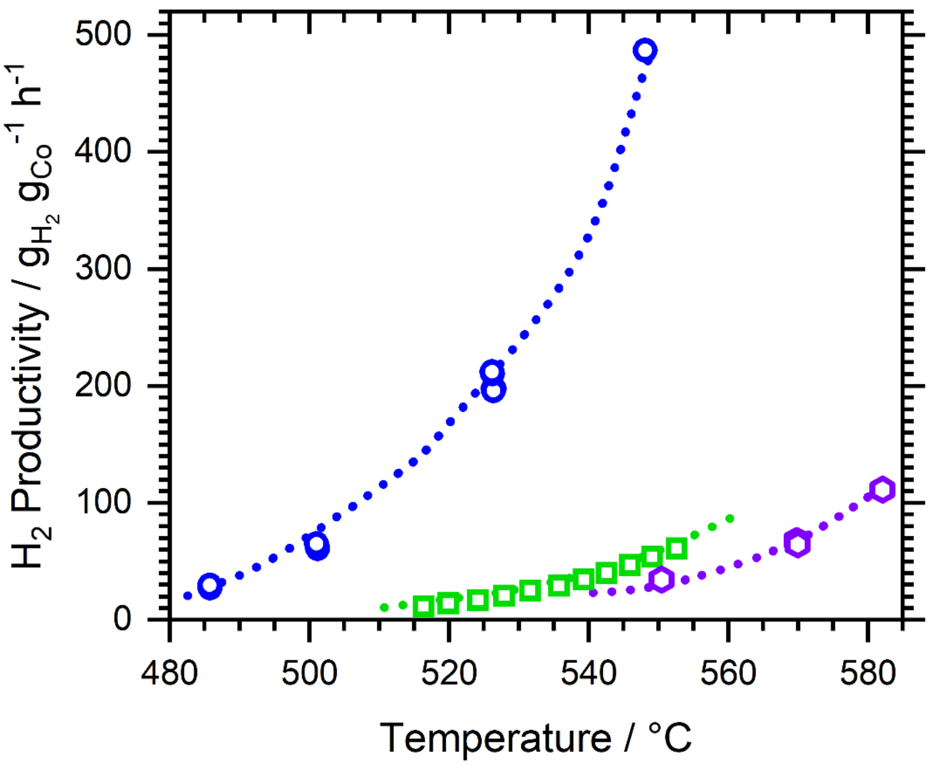

Figure S 11: H_2 productivity specific to the Co mass at 5 ± 0.5% NH_3 conversion at different catalyst temperatures for $Ga_{59}Co/SiC$ (blue circle ○, m_{cat} = 1.00 g, V_{cat} = 1.8 ml, w_{Co} = 0.04 wt%, w_{Ga} = 3.12 wt%, WHSV = 2-29 $l_N\ g_{cat}^{-1}\ h^{-1}$, p_{total} = 1 bar(a)), 1.05 wt% Co/SiC (light green square □, m_{cat} = 0.25 g, V_{cat} = 0.73 ml, w_{Co} = 1.05 wt%, WHSV = 8-58 $l_N\ g_{cat}^{-1}\ h^{-1}$, p_{total} = 1 bar(a)), and 0.05 wt%Co/SiC (purple hexagon ⬡, m_{cat} = 1.00 g, V_{cat} = 1.5 ml, w_{Co} = 0.05 wt%, WHSV = 3-9 $l_N\ g_{cat}^{-1}\ h^{-1}$, p_{total} = 1 bar(a)). Dotted lines to guide the eye.

Stabilisation and saturation of $Ga_{59}Co$ SCALMS

Figure S 12 shows the deactivation of a $Ga_{59}Co$ SCALMS catalyst over 90 h of constant ammonia flow beyond 247 h TOS, as indicated by changes in NH_3 conversion and the corresponding H_2 productivity specific to the Co mass. After an initial flushing with H_2, the system was maintained at a constant volume flow of 10 L_N min^{-1} at 579 ±1°C. The system showed a conversion of 11.7 ± 0.0% at 247 h TOS, followed by a steady deactivation (deactivation rate 0.2 % h^{-1}) to reach a conversion of 9.5 ± 0.0% after 12 h. The slope then steadily decreased, stabilizing at a conversion of 8.4 ± 0.0% after 30 h (277 h TOS). We attribute this curve profile to a classical adsorption system that saturates over time; in this case, water is adsorbing at the SCALMS system. A breakthrough of water could not be observed because the water content of the pure ammonia was below the detection limit of the analytics used.

The H_2 productivity of the system decreased from initially 357 g_{H2} g_{Co}^{-1} h^{-1} to 257 g_{H2} g_{Co}^{-1} h^{-1}.This corresponds to an activity loss of approximately 30 %. The activity of the SCALMS system remains significantly higher than all activities observed for monometallic Co systems. This highlights the fundamentally different nature of the SCALMS system compared to monometallic systems.

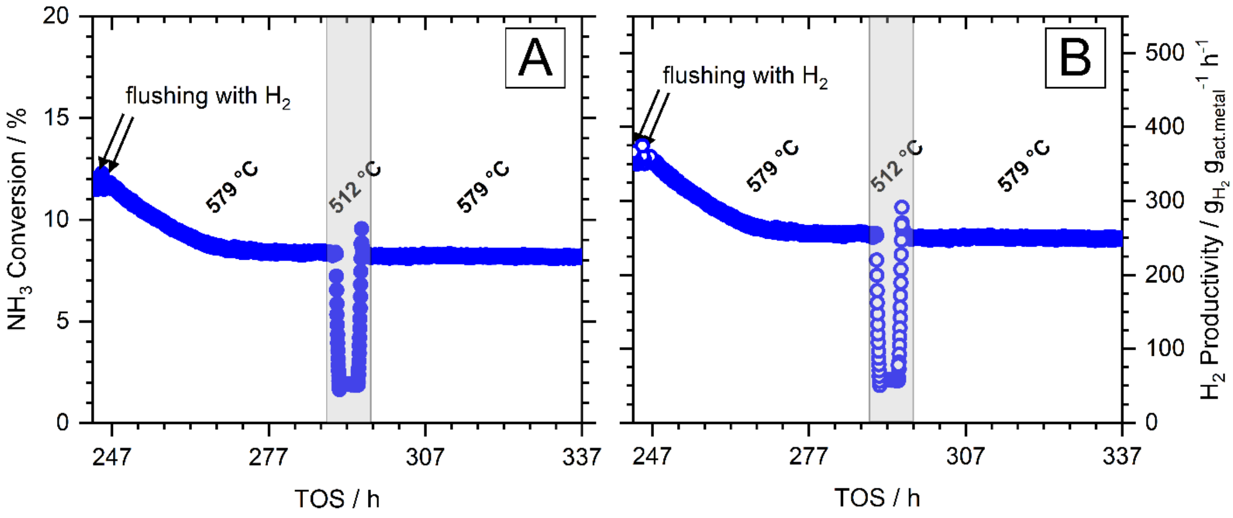

Figure S 12: NH_3 conversion (Figure S 12A, <u>filled symbols</u>) and H_2 productivity specific to the Co mass (Figure S 12, <u>hollow symbols</u>) over time on stream (TOS) for $Ga_{59}Co/SiC$ SCALMS (blue circle ●, m_{cat} = 1.00 g, V_{cat} = 1.8 ml, w_{Co} = 0.04 wt%, w_{Ga} = 3.12 wt%). Reaction conditions: T_{cat} = 579 °C, p_{total} = 1 bar(a), WHSV = 10 l_N g_{cat}^{-1} h^{-1}.
